# Dr. Laycock on Mind and Brain

**Published:** 1860-07-01

**Authors:** 


### Art V.—DR. LAYCOCK ON MIND AND BRAIN.

Dr. Laycock has very happily supplied a great need in English medical and psychological literature. In the work noted below* he has discussed the theoretical and practical questions of psychology and biology with a breadth and acuteness rarely equalled. But the matter of most immediate interest in connexion with this book is, that it is designed—"*first*, as a class-book, to introduce the student of medical psychology to a comprehensive inquiry into the relations of consciousness to organization; *secondly*, to afford to the general student of mental science, in its practical applications, whatever these may be, a solid foundation for a course of self-culture, in an exposition of the relations of organization to consciousness."—(p. vii.)

Simply as a class-book Dr. Laycock's work would fill up a very ugly hiatus in the psychological, and particularly in the medico-psychological literature of this country. But the book possesses, for us, even a much higher interest than this. For we would fain hope that it will contribute in no small degree to bringing about the establishment of specific tuition in medical psychology in our schools of medicine generally, as it most assuredly removes one formidable obstacle to that tuition, to wit, the often much-felt want of a trustworthy text-book for the student. We hope also that this work will not be without effect in inducing our anatomical and biological lecturers to place their teachings on a much higher footing than is customary. For Dr. Laycock's admirable exposition of the true practical value of a thoroughly philosophical biology must commend itself to all who are interested in biological science. Indeed, apart from its interest as a class-book for students, the work is of high scientific interest as containing the evolution of a philosophical system of rare attractiveness. It might, perhaps, be said by some, that much of the material of which the book is composed is too concentrated, too close and firm of texture to suit well the unsophisticated mental stomachs of babes and sucklings in psychology and biology. But there is this great consolation, that any, even the most petulant babe *may*, if it will, take the nutriment, and that those babes who are reared upon it may look forward to a very healthy and vigorous manhood.

Dr. Laycock's work will be more justly dealt with by description than by criticism, and for this reason:—The subjects he

* *Mind and Brain: or, the Correlations of Consciousness and Organization; with their applications to Philosophy, Zoology, Physiology, Mental Pathology, and the Practice of Medicine.* By Thomas Laycock, M.D., F.R.S.E., &c., Professor of the Practice of Medicine and of Clinical Medicine, and Lecturer on Medical Psychology in the University of Edinburgh. 2 vols. Edinburgh.

deals with are open to considerable difference of opinion, and it is not so much our agreement with or difference from this or that opinion which he expresses which should guide us in our approval or condemnation of the book, but our decision should be founded upon the mode in which we conceive the chief object of the work is attained, to wit, as a class-book for the medical and general student. Two considerations, we apprehend, should principally govern us in deciding upon this point: (1) the *sound-mindedness* (to use a phrase of Dr. Whewell's) of the doctrines taught in the book, and (2) their probable effect upon the ulterior study of the subjects to which they refer. Now a general description of the scope and argument of the work will, perhaps, best show in what degree Dr. Laycock's teachings possess sound-mindedness,—the first requisite we have named; also, in what degree they are calculated to foster psychological and biological studies,—the second requisite.

Dr. Laycock prefaces his work by stating, that after long research into the relations of body and mind, he had been led to the conclusion that the resources of mental philosophy and cerebral physiology bearing upon the inquiry were vitiated by a fundamental error in method. "Our investigations concerning mind and brain neither attempted," he writes, "nor indeed pretended to attempt, a demonstration of the relations between the laws of functional activity of the organ of consciousness and the laws of consciousness itself. Each department of knowledge was expressly held apart from the other; and their subject-matters of inquiry were considered to be fundamentally distinct." He then proceeds:—

"It is easily seen why this divisive method of inquiry is not applicable to practical ends, either in mental or vital science. Since we have no other sources of knowledge than those which our consciousness affords, and since all our states of consciousness are necessarily coincident with the operations of our vital forces, it follows, that a knowledge of the laws of consciousness, in relation with the laws of the vital forces, must constitute the foundation of all science whatever; or, in other words, that metaphysic, to be complete as a unity, must bring within its range the laws of life and organization.

"The union of philosophy proper with physiology (or, more correctly, with biology) is, however, more especially necessary, if we would establish an applied science of mind; for that must be applied to mind active in all the business of human life, whether in the healthy states of which the metaphysician takes exclusive cognizance, or in those morbid states which more especially engage the attention of the physician. In both classes of mental activities the phenomena of life and consciousness are inseparable; so that, without a scientific correlation of the two classes of laws, an applied science of mind is not possible."—(p. v.)

In accordance with these views Dr. Laycock attempts an introductory exposition of the correlations of physiology and philosophy in the work before us, seeking to adapt it as a class-book for the student of medical psychology and for the general student of mental science (as we have already stated). The scientific physician, the advanced metaphysician, and the philosophical phrenologist are pretty well agreed that the construction of such a work is among the scientific demands of the age. Moreover, the perplexing social questions constantly arising in reference to insanity, teach us the absolute necessity of possessing a more practically available science of mind than we yet possess, if we would legislate beneficially on these questions. Again, in "a mental philosophy which takes due cognizance of the laws of life," will be found the best antidote to those pseudo-scientific abominations, mesmerism, electro-biology, and the like, which haunt the highways and bye-ways of biological and psychological studies, seducing many innocents to destruction. Further, by a scientific correlation such as that indicated, we can alone hope satisfactorily to solve the oft-disputed questions which so often bewilder the student in natural history, the zoologist, and ethnologist, that is to say, the difference between instinct and reason; the limits of animal and vegetable life; the origin of living things and of man upon the earth; the differences of race, form, and mental qualities among men. Nay, the correlations of the laws of organization and consciousness must be the next great step towards the foundation of a science of social organization.

So widely would bear a successful application of our author's opinions, and in endeavouring to effect this, he has "sketched a method, and carried it out into the formation of a body of doctrines, in such a way that the reader, if an earnest student, may have a guide through the multitudinous phenomena he has to examine and compare, in his scientific progress from the known to the unknown."—(p. xi.)

In the first part of his work Dr. Laycock sets forth the necessary connexion between physiology and mental philosophy, and states his method of inquiry; in the second part he gives a summary of the results of experience, and of the general doctrines reached by speculation and the divisive method; in the third part, which commences the scientific portion of the work, he discusses the causes of life and consciousness. He writes:—

"Of late years science has developed the unity of the physical forces, and reduced them to a general law of transference of force; the correlations of the physical and vital forces in this respect have also been worked out. And no one has shown how forces are transferred so as to attain the ends which are observed to be attained by the operation of the vital forces; nor has any one attempted to throw a scientific bridge across the impassable gulf which has hitherto appeared to separate the phenomena of life and organization and of thought. Now the author has aimed to overcome this difficulty by a new and very simple method, and one perfectly available, as he believes, for all purposes of inquiry. Looking at the two classes of phenomena, and examining what they have in common, this principle is deduced—viz., That whereas mind designs, life is designed. Design, therefore, is common to both; but in the one there is a conscious energy of design, in the other an unconscious. And this further law of correlation is universally manifest—viz., That the results of the vital forces, operative according to a law of design, coincide with the various states of consciousness known as desires, feelings, and the like. Hence a general law of design, with its derivative laws, correlates both the laws of life and of consciousness." (p. xii.)

In the third part, also, Dr. Laycock developes the principles of teleology, or mental dynamics, from this law of design; "and Ideas are considered as causes not only of life and thought, but of all the phenomena of creation." This portion of the work Dr. Laycock anticipates will probably attract more critical attention than others. He desires, therefore, that it should be understood that—

"— the views therein developed are intended to be wholly scientific. Mind is simply considered as an ordering force in creation, to be examined according to the usual method of scientific research; that is to say, as it is manifested in the sequences and co-existences of phenomena. There is no discussion as to the nature of soul, mind, or spirit, such as is found in psychological works generally; and thus the phenomena are examined wholly apart from those philosophical and theological speculations which are altogether foreign to science. In introducing, therefore, so much of these speculations as is to be found in this part, the author had solely in view the restriction of the inquiry within its proper limits." (p. xiii.)

Dr. Laycock having thus established a system of general principles, proceeds to apply them in succession to the general laws of biology, the development of a scientific cerebral psychology, and to the first principles of a mental physiology and organology, always keeping in view the solution of practical questions, or the suggestion of new methods of inquiry. Finally, Dr. Laycock writes, that:—

"The entire scope of the work is to carry up the doctrine of final causes in a connected form, to its highest uses; and to show that Mind is the final cause, as an ordering force, of all the physical forces, and of all their derivative manifestations in the phenomena of creation. Under the guidance of this principle, that union of the two great departments of human knowledge, hitherto so sedulously kept apart, is attained. Thus, the work it is hoped may serve to advance both; for on the one hand the phenomena of life and organization are brought into the domain of philosophy; on the other, the phenomena of thought are brought into the domain of physiology. The unifying principle, that mind is dominant over matter and its forces, enables us to compare and generalize phenomena hitherto considered wholly discordant, so as to harmonize them, and thereby to break through that eternal maze of contradictions, as to reason and instinct, consciousness and unconsciousness, will and intelligence, within which all philosophical inquiry has been so long involved. By adding physiology to philosophy, we place philosophy at the head of the inductive sciences, and at the same time bring all the sciences of life and organization into philosophical relation and unity. The basis of this unity is teleology, applied deductively and inductively to all the phenomena which science investigates." (p. xv.)

Such is in brief the argument and scope of Dr. Laycock's work as gathered from the preface. We turn now to the body of the book.

In the Preliminary Dissertation on Method, which forms the first portion of the work, our author discusses first the necessary connexion of a physiology of the brain with a practical science of mind. If we would know, he argues, the laws and modes of existence of man as a rational being, it is needful we should know "not only how the encephalon is constructed mechanically, and how it acts dynamically, but under what conditions it is constructed, and according to what laws it is operative." In other words,—

" . . The laws of development and organization of the encephalon, as the necessary instrument and seat of man's energies and feelings, must be determined, if we would rightly understand its mechanism and modes of activity, under varying states of the consciousness, in various races, nations, societies, individuals. The vital processes and the correlative mental states of each moment are linked to a series of similar changes, of which they are logically the necessary results, extending far back into time. This long series of activities—vital and mental—continuing through a succession of individual men, from generation to generation, corresponds, in cerebral physiology and psychology, to that long succession of forms of organisms which, commencing in the abysses of time, are the subject matter of palæontology; so that just as the last individual of a species is but the latent expression or manifestation of a long series of cycles of changes in the species, so the last thoughts, and feelings, and actions of an individual man are but the result of a series of changes dating far back in his own individual history, or having their roots in the modes of mental and vital activity of his ancestry." (p. ii.)

Now, it is not to be denied that throughout these cycles of vital changes an "immanent, inherent energy" is ever operative—an energy which—

" . . . Is not a more physical or material agent, and which can only be conceived as an actively adapting force manifested in the phenomena of life; consequently, its laws and modes of operation can only be determined by determining the laws of development and organization, and of mechanical structure and dynamical action of the living structures by which it is manifested. If it be inoperative, it is unknowable. Hence, mind and its laws can only be known through the phenomena of life and its laws. And since the phenomena of human consciousness are biologically a special form of manifestation of vital processes, which have their seat and origin in the encephalon, the laws of thought, and will, and feeling, can only be fully determined for practical uses in correlation with the laws of action of the nervous system, of which the encephalon is the chiefest portion and the highest development. It follows, therefore, from these considerations, that the brain and nervous system are the proper subject matter of a true science of mind —without a knowledge of which there can be no such science whatever, in the proper sense of the term. It equally results, that its study as an applied science can only be followed according to the method pursued in the study and application of the other applied sciences." (p. 3.)

Hence, then, the study of the correlations of the laws of action of the brain and nervous system with those of thought and volition must hold the chief place in our researches for the establishment of a sound science of mind. Apart from such study, we must altogether avoid attempting to frame a science of mind by simple meditation on our own states of consciousness, or by the exercise of logical art. "No one," Kant has observed, "by means of logic alone can venture to predicate anything of or decide concerning objects, unless he has obtained, independently of logic, well-grounded information about them." And we must not run into the error, which has been committed by many modern inquirers, of neglecting or treating as of secondary importance either logic or metaphysics. Both are of great importance in facilitating and fostering close and accurate observation of phenomena, and Dr. Laycock well remarks that "the most successful inquirers have usually been good metaphysicians."

The attainment of the knowledge which our author instructs us we should strive for, if we would construct a true science of mind, is beset by sundry difficulties. These difficulties, however, do not belong so much to the knowledge itself as to the faulty methods which have been employed to reach it. To guard the student from these erroneous methods and to establish a better method are the chief objects of Dr. Laycock's Preliminary Dissertation.

Now, while a true method of research is necessary to and would facilitate the acquisition of a sound science of mind, and while it may be assumed that the study of mental science "is not only the most attractive of all scientific pursuits, but also that the science itself is easy of acquirement, and much more within the reach of the general public than those which require elaborate apparatus for their successful prosecution,"—each student having in the phenomena of his own consciousness both the means and the matter of observation, his own brain being an instrument which, if rightly used, exceeds all other instrumental aids to research whatever (p. 6),—still it must not be imagined that either the successful study or the practical application of mental science is possible, without the mind has been previously disciplined in habits of observation. Dr. Laycock adds to his introductory observations the following just caution :—

"Although I am anxious to popularize the science of mind, and therefore seek to break down the barriers which have hitherto excluded the great majority of educated men from its pursuit, I only mean it to be popular in the sense of being non-professional, and not popular in the sense of its being a thing easy of acquirement. The student owes scientific labour and serious thought to it as a sacred duty; for without these mental science will degenerate into a superficial, frivolous pretence of knowledge. The true psychologist, therefore, must be ready himself, and demand of others, to submit to discipline; must toil to acquire the necessary preliminary knowledge which may be considered only instrumental, as the anatomy and physiology of the nervous system, and the sciences of life and organization, so far as regards general principles; must acquire a precision in the use of terms as nearly of mathematical exactness as the ambiguities and imperfections of language will allow, together with that skill in the use of his intellectual powers which a logical training supplies; and finally, he must practise his observing powers in his daily intercourse with man, and not with man alone, but all the living things his fellow-creatures about him, with a special reference to the great object of his studies. Now, if this list of requirements may appear in imagination so great as to dishearten the inexperienced student, or the man who has been accustomed to limit his investigations to the phenomena of his own consciousness, I would just remark, that the reality is by no means so overwhelming as the imagination. On the contrary, I think that a practical science of human nature may be successfully studied in equal time and with equal ease as most of the higher departments of other sciences, as for example the higher mathematics, physical astronomy, chemistry, engineering physics, and the like, provided always that the same method and the same perseverance be used in acquiring the practical science of human nature as in acquiring them." (pp. 10, 11.)

Dr. Laycock next proceeds to discuss the ends to be attained by a science of mind founded on a philosophical physiology of the brain. A practical science of mind, he holds, "must be capable of a threefold application to practical ends : namely, first, to advance the happiness of man in his individual and domestic relations; secondly, to secure the welfare of society; thirdly, to advance that knowledge which secures the development of the race, and establishes man's dominion over nature. These form an ascending series of objects necessarily in relation to each other, and I will endeavour to illustrate each series. One and all, however, can only be aimed at through a mental science based on a philosophical physiology of the brain."—(p. 15.) Dr. Laycock then briefly shows the applications of mental science to mental hygiene, medical art, medical science, professional pursuits, sociological psychology, the psychology of public hygiene, and of climatology and ethnology. He also points out the dependence of social progress on mental science, the psychological relations of agriculture, the connexion of national mental vigour with healthy physical conditions, the relationship of the fine arts to a vigorous national life and sound morals, and terminates an admirable outline of these important subjects with a sketch of the relations of mental science to a complete philosophy, finally remarking:—

"These, then, are the great objects of a full and complete mental science, in relation with a philosophical physiology. It is co-extensive in its scope with created things. It investigates the action of the physical forces, that it may determine their relations to the forces of life and organization; it investigates the vital forces, that it may develope the relations of life and organization to mind; and it investigates the phenomena of mind in their relations to physical and vital forces as operative in the brain, that it may deduce general principles applicable alike to the material welfare and the highest moral, intellectual, and spiritual interests of man. It is necessarily co-extensive with that knowledge in which all men take interest, and is synonymous with that philosophy which Kant defined as the science of the relation of all cognition to the ultimate and essential aims of human reason (*teleologia rationis humanæ*)." (p. 32).

Assuredly this is a wide-reaching scope; but Dr. Laycock fully justifies this summary and deprives it of all aspect of exaggeration in the subsequent portions of his work. He conceives most nobly of his subject, and his execution befits well the nobility of his conception.

Our author next examines those obstacles to the development of a practical science of mind which arise out of the adoption of erroneous principles and imperfect methods; and pursuing the subject still further, he discusses the restrictions on the progress of mental science, from the prejudices of mankind and the dogmas of speculative metaphysics and theology. This subdivision of the work will prove of great value to the student and general reader; and the latter portion is distinguished by a catholicism, which, together with the practical tendencies of the work already set forth, at once sets at rest the *sound-mindedness* of Dr. Laycock's doctrines. Treating of the prejudices arising from the apparent antagonism of Mental Science to Revealed Truth, he writes:—

"We cannot but think that Bacon, and other philosophers of his day, were not too enthusiastic when, contemplating the grandeur of modern science, they earnestly expressed their belief that these are the days referred to by one of the Jewish prophets as those in which 'many shall run to and fro, and knowledge be abundantly increased; and the knowledge of the Lord shall cover the earth as the waters cover the sea.' Science itself, in its highest and fullest development, is religion; for although there may be now and then an 'undevout astronomer,' yet the deepest thinkers are agreed, and have always held, that a knowledge of creation and its laws can only lead to the knowledge and love of God." (p. 84.)

The objects of a practical science of mind having been examined and the obstacles to the attainment of such a science ascertained, the next point is to determine what are the principles and methods which would enable us most readily to avoid these obstacles, and to develope best the science sought for. Having first shown that all the fundamental principles of a practical philosophy must be deduced from the experience of mankind, corrected by enlightened observation and inquiry, Dr. Laycock passes on to examine the method of determining the first principles of a science of mind by observation and induction, and finally concludes that the teleological method is the most natural and universally applicable method. He joins issue with Dr. Whewell, one of its ablest advocates, in restricting the application of this method "as a fundamental and regulative idea to our speculations concerning organized creatures only," and doubts whether the authority of Bacon, as opposed to the teleological method, has been rightly quoted. Our author points out also the imperfections which have too often vitiated the applications of the method, and shows that the principle of *final* unity is that alone which guides us teleologically. Using the teleological method rightly, we are to seek to "discover the Divine counsels, so far as that is possible, by observing the ends attained, and classifying them; or in other words, determine the results of uniform successions, co-existences, and the like. We should thus be enabled, while we were determining these results, to determine the laws of mind, considered as an ordering force in creation. From this point of view, teleology would be something more than a questionable doctrine of 'final causes,' inasmuch as dealing with Mind as an ordering force, it would be strictly the science of *Mental Dynamics*." The objects aimed at are not, however, to be attained solely by the extension of the teleological method. This indeed will fail us, unless it be so developed that the principles educed by it are demonstrably conformable with both the inductions of science and the experience of mankind. "In other words, those principles must be subjected to the double proof required to establish all general principles whatever." (p. 113.)

Dr. Laycock having thus determined upon a method of research, advances next to its practical development. And, first, he institutes an inquiry into the general and scientific experience of mankind as to their states of consciousness (Empirical Psychology); next, he examines into the fundamental laws of existence (Ontology); and, thirdly, he discusses the first principles of Mind as an ordering force to ends (Teleology, or Mental Dynamics). The tendency and scope of these three great divisions of his work are thus summed up by Dr. Laycock:—

"In the first we examine Consciousness in relation to vital phenomena; in the second, Existence in relation to vital and physical phenomena; in the third, we develope the great correlations of Mind with the physical and vital forces considered in relation to design in creation, viewed as a systematic unity, or the doctrine of Ends. This will bring the hignest manifestation of mind—as a creative and regulative power—into synthesis with creation, and consecutively into synthesis with the human mind. Here, the method will show that the ideas of the Divine Mind, as revealed in the phenomena of creation, are none other than the fundamental ideas and *à priori* conceptions of the human mind as revealed in consciousness; that the ends aimed at and attained by the Creator are the objects of the instinctive desires of the creature; and that, consequently, the phenomena of nature constitute a reflex of the human mind. Or, to use the words of M. Agassiz, 'the whole may be considered as a school in which man is taught to know himself and his relations to his fellow-beings, as well as to the First Cause of all that exists.' In this way we shall have completed the task which we proposed at the outset—namely, to develope a method of philosophical inquiry which should combine the three great departments of human knowledge into unity, and attain to a knowledge of human nature, not empirically only, but deductively, through principles derived from the entire range of all science." (p. 114.)

We shall not attempt to follow step by step the development of our author's method as thus stated, but simply cull a few illustrations which may suffice to mark some of the more important stages of the development. And, first, as to the relation of consciousness to vital phenomena. "Since Descartes limited psychology to the domain of consciousness," writes Sir William Hamilton, "the term *mind* has been rigidly employed for the self-knowing principle alone. Mind, therefore, is to be understood as the subject of the various internal phenomena of which we are conscious, or that subject of which consciousness is the general phenomena.'"* Now, if we seek to ascertain the relations of mind with matter, or rather with the forces of matter, with which we can legitimately institute a comparison, we are led to the conclusion that "mind is that which has the power of beginning motion; matter has not the power: mind is that which feels and thinks; matter does not feel or think: mind adapts events to designed ends; matter is adapted to ends: mind is conscious, matter is unconscious; or, finally, since all these are included under consciousness, mind is consciousness." Upon this conclusion of the nature of mind in comparison with matter several important, and perhaps the most currently-received, systems of modern philosophy are based,—the forces of matter and mind being held wholly apart. But is this disjunction consistent with experience, or necessary to a successful investigation of mind? To determine these points renders requisite an inquiry into the relations of consciousness to Existence or Being. "If Beings manifest all the phenomena we attribute to mind, although not conscious, then mind must be the cause of being, and therefore of something more than consciousness." (p. 124.)

Now, if we attempt to draw a line *anywhere* between vital and psychical forces, we shall most assuredly be foiled. Mr. J. D. Morell, adopting the doctrine of experience current in Germany, that continuous consciousness is not necessary to being, the soul being *prior* to consciousness, has admirably said that,—

"Even in the early unconscious developments of *life*, there is an intelligible purpose manifested which denotes the presence of a rational principle, although that principle only manifests itself as yet in teleological forms and processes. *Instinct* again plainly betokens mind, only in a lower sphere. . . Neither is it possible, if we go one step further, to separate the phenomena of *sensation* from those of the physical and vital forces. The conscious and the unconscious sides of the process are so blended together, that it is only by a mental fiction that we distinguish them, and assign a cause to the one different from that which produces the other."

And if we go upwards to the higher intellectual regions, even to the will, still these are only reached—

"By a succession of steps, all involving both thought and feeling, between no two of which we can draw any line of demarcation, so as to say where the vital and automatic processes *end*, and where those of the soul, *par excellence*, begin. The whole, in fact, are so interwoven in producing the result, that they point as of necessity to a primitive unity as the real starting-point of them all."†

Dr. Laycock concludes, therefore, that—

* *Lectures on Metaphysics*, vol. i. p. 129.
† *Elements of Psychology*, part i., p. 76.

"It is thus clearly deducible from experience that consciousness cannot be separated as to causation from existence or being. Existence is certainly implied in consciousness, but is not dependent upon consciousness. Life and mind are correlative in consciousness, and dependent, therefore, upon correlative forces: Knowing and Being have the same cause." (p. 130.)

Passing over Dr. Laycock's interesting summary of the general doctrines of consciousness, we reach the ontological division of his book, and we cannot resist the temptation to quote the following characteristic paragraphs from the chapter on "Unconscious Existence:"—

"We cannot conceive how mind could *act* independently of matter, although we can conceive it to *exist* independently of matter. But the conceivable in thought, and the conceivable in fact or act, are wholly different things. Thus the geometrician defines a point to be that which has neither parts nor dimensions. Can we, however, by any effort, realize a point—that is, an actual thing without parts or dimensions? The nearest conception of some would certainly be the idea they had formed of soul. Then as to causation, which is involved in our conception of mind, we can conceive a cause in the abstract, but can we realize it without at the same time conceiving the thing caused—the effect? This is a matter of experience. I must say that I cannot. How can we comprehend Mind as a cause of motion until we know that motion results from its operation? or how can we comprehend it as manifested, before any manifestations of mind appear? All abstract truths end thus in the inconceivable—just as all conceivable time ends in infinite time, all space in infinite space, and the like.

"Now, we *can* conceive mind to be existent and *latent*,—that is, if manifested in a past time, as not manifested *now*, or if manifested now, as not to be manifested at a future time. In this way we arrive at the conception of the latent existence of the soul after death—that is, as not manifested *for a time* until the man lives again; or, in other words, until the mind is manifested in another body. And this process is the more easy, because we already apply it to the comprehension of those powers or forces of matter, that, like mind, are known only by their effects. We thus speak of *latent* heat; that is, heat not manifested by changes in matter, or in our own bodies. The so-called electric and magnetic fluids may in like manner be latent; that is to say, only manifested when matter undergoes a change cognizable by us. It is, indeed, on these relations of the "Imponderables" to matter, that the doctrine of a correlation of forces is founded. As we shall speedily see, what we call light, heat, magnetism, electricity, chemical affinity, &c., are the manifestations of one and the same primary force acting differently under varying conditions, but which are only possible in and through matter.

"Applying these illustrations to mind in its relations to matter, we clearly see that the generalization which distinguishes between mind and matter, is as well-founded as that which distinguishes between matter and the forces of matter; but it is equally true that we can no more realize mind as acting apart from matter, than we can realize the force of gravity or of chemical affinity as acting apart from matter. And this is one of the great empirical laws derived from human experience. The term *individual* indicates the one indivisible being constituted of both matter and mind. No man of common sense believes that his mind really acts in the ordinary concerns of life, *does* anything in the world apart from his body. So also as to the laws of society. In the entire decalogue, nothing else is referred to but the man. No human laws give human rights to the dead; they are no longer men, but 'souls' only, and cease therefore to be members of human society. It is quite remarkable, indeed, how often the common sense and experience of men triumph over man's most cherished and deep-rooted superstitions. Of the great majority in the United Kingdom who believe materialistically in ghosts, how few act up to their belief!"

Further, Dr. Laycock rightly observes that the true source of all the discussions as to whether consciousness is continuous or not, is in the fundamental doctrine which holds mind apart from life.

"When we reverse the doctrine, and include life under mind, as the common cause of both conscious and unconscious existence, we view consciousness and unconsciousness in the same light as they are placed by the experience of mankind—namely, as modes of existence which are determined by the varying phenomena of vital action. Such being the general law, the proper inquiry is, what vital phenomena correspond to conscious states of existence, what to unconscious. These are the phenomena of conscious and unconscious cerebral action." (p. 173.)

Dr. Laycock now examines the question of latent consciousness, following closely the views of Sir Wm. Hamilton, and next he enters upon the examination of instinctive experience. Looking upon the term instinct in its widest application, "as manifesting a blind, unconscious adaptation to ends," he would class under the term instinctive the actions appropriate to nutrition or alimentation, and of respiration or aeration of the tissues. "Hence instinct, in this more general sense, is a property of vegetable organisms as well as of man. It is, therefore, the supposed cause of all those acts which are performed either without any mode of consciousness absolutely—that is, unconsciously: without any knowledge on the part of the individual, of the ends to be attained by the acts, or without any volition." Thus Dr. Reid: "He [a new-born child] is led by nature to do those actions [of sucking, swallowing, &c.] without knowing for what end, or what he is about. This we call *instinct.*" Now we can hardly find more suitable expressions, as Sir Wm. Hamilton has said, "to indicate those incomprehensible spontaneities of which the primary facts of our consciousness are the manifestations, than *rational* or *intellectual instincts;*" and it will scarcely be doubted that, as Dr. Laycock remarks, "the same energy which acts as instinct, and is esteemed a *quoddam divinum* in lower organisms, is identical with the energy acting as instinct, and termed soul in man."—(p. 192.) If we push our analysis still further we are irresistibly led to the conclusion that the difference between the mental nature of men and animals is one of degree only, not of kind,—thus we arrive at a position from which we can generalize thought and instinct under one term, with this common to both,—"that each is the manifestation of a law of necessary adaptation to ends."—

"The man ceases to be rational when he aims not at an adaptation to ends. Instinct, we have seen, is that adaptation itself. In trying to realize from the side of instinct a more general conception of *soul*, or *mind*, we find that it contains three elements. 1. There is the *law* of necessary adaptation to ends. 2. There is the pure reason or thought, the '*quoddam divinum*,' by which the ends are conceived, and the machinery and methods for attaining them designed. 3. There is the force or energy which is manifested in and by the law of necessary adaptation to ends, and which is active or operative in accordance with the conceptions of the pure Thought, so that the machinery necessary to attain the end is constructed out of matter, and worked appropriately when so constructed. Now, these three elements enter into the intellectual as well as the instinctive nature of man. A fourth needs to be added, *i.e.*, consciousness, as the common characteristic of all. Consciousness of adaptation to ends, or Thought; consciousness of the successive events necessary to secure adaptation, or Knowledge; consciousness of the exercise of the power or energy used in the actual adaptation, or Will; and, finally, the *feeling* associated with the processes—the Desire to attain an end, and to adapt to the attainment; the Desire to attain the knowledge necessary to secure the adaptation; the Desire to exercise the power necessary; and, finally, the Pleasure or Pain consequent upon the attempt at adaptation with the success or failure." (p. 195.)

We may now infer the differences between man and animals; but between the highest and lowest capabilities of knowledge, both in man and animals, so unbroken is the series that we can nowhere strictly draw a line of demarcation and say, Here instinct ends, here reason begins.

Dr. Laycock's reasoning upon instinctive existence culminates in the following generalization:—

"It follows, then, from all these considerations, that all those general phenomena of mind which we have already investigated are instinctive in their character; or, in other words, are due to the same cause or causes, and occur according to the same laws, as the mental phenomena of lower animals. Now, these phenomena are vital; consequently, the mental phenomena of man are vital.

"By successive generalizations of the facts and propositions of experience, we thus arrive at the ultimate generalization attainable, and the phenomena of life are brought under the same efficient cause—a spiritual Energy—as the phenomena of mind. Nowhere can we draw a line of demarcation between these phenomena as to causation, so that the soul, considered as an Energy, acts equally in nutrition, development, and instinct, in all states of consciousness, and in all intellectual operations whatever. These phenomena are but varying manifestations in time and space, through matter, of the same spiritual thing. *Soul* operates in all. So that, by the operation in matter of the 'immaterial *Ego*,' according to the necessary law of adaptation, vital force and nervous force, instinct or blind adaptation, and reason or conscious adaptation, are variously developed into activity, as modes of action of the same force, according to that law of adaptation itself; and thus the vital and the mental forces and functions are but manifestations of the same pure thought in action. This generalization is exhaustive, being co-extensive with all the phenomena of creation, and is the only generalization which can comprehend both the phenomena of Life and Mind." (p. 197.)

We shall not dwell upon Dr. Laycock's observations on speculative ontology, which follow next in order. One remark, however, we may make before entering into the third sub-division of his work, to wit, that all systems of speculative phenomena have had running throughout them the great fundamental truth, that "Mind is the final cause of order in creation." (p. 212.)

Having in the ontological sections of the work discussed the question of existence in relation to vital and physical phenomena, Dr. Laycock proceeds to apply the principle of teleological unity which he has arrived at, and which Kant has designated the highest of the principles of unity, to an examination of the "innermost secrets of nature" lying hid in the relations of mind to the phenomena of life and thought, and to the development of the laws of mind as the cause of all phenomena. This constitutes the third sub-division of his work, "Mental Dynamics, or Teleology."

And, first, he treats of the general doctrine of the correlation of causes, and passing briefly under survey the general phenomena of force and life, he concludes that, "mind is the universal, necessary, unchanging, causal element in all our cognitions of ourselves and of the universe; matter, as represented by the physical and vital forces, being the contingent, variable, ever-changing, causal element. Mind is the *First Cause;* the one absolute thing, from which all other causes and things are derivative." (p.

226.) The next step in the development of the method is to determine "the correlations of life and consciousness, through the correlations of mind with the physical and vital forces, as displayed in the unity of created things." In pursuance of this object Dr. Laycock passes under review, first, the doctrines of the correlations of the physical forces, and next those of the correlations of the physical and vital forces. He remarks that—

"In the modern doctrine of the correlation of the physical forces, the unifying idea is that they are all convertible into motion, and that they are simply manifestations of the different relations to each other in which atoms are placed. But there has been no teleological development of the doctrine in its application to Life and Thought. Force has been simply personified according to the ancient and vulgar plan." (p. 251.)

In illustration of this view Dr. Laycock quotes the opinions expressed in the well-known paper by Dr. Carpenter, "On the Mutual Relations of the Vital and Physical Forces."*

Dr. Laycock then proceeds to examine the correlations of the physical, vital, and mental forces, and having shown, "as a logical necessity, that a law of design comprised all those fixed unvarying successions of events which we attribute to the physical forces," also that a similar law comprises all the manifestations of organic forces, he concludes that "design is as much a part of the phenomena of creation as force, law, or uniformity. Each correlates the other."

"Further, if the vital forces be derivative of the physical forces, which we may now accept, temporarily at least, as a truth in science, it necessarily follows that the law of design which characterizes them must be derived from the physical forces: and if it be admitted that there be but one universal and absolute, then it is logically necessary that Mind, as manifested by a law of design, is that universal and absolute. Again, in no other way can we conceive mind as being absolutely universal—that is, both in the relative universal and in the particular—than by the doctrine that it is the universal of the physical forces of creation in our cognition of force, of which vital forces are the variable, contingent, and derivative. Again, a mental force must have a correlative law; now that correlative law in creation is a universal law of design." (p. 264.)

Our author next enters upon an investigation of ideas as causes, governing his method of procedure by the propositions that, "inasmuch as no state of consciousness whatever happens without correlative vital processes, so it follows that no vital processes, whatever they be, or however they be named, happen independently of their correlative ideas and intuitions of mind in

* *Philosophical Transactions.* 1850.

the abstract."—(p. 271.) Discussing first the general doctrines of Ideas as causes, Dr. Laycock then examines the modes of Derivative Evolution of Ideas so regarded, and the classification of these Ideas. Further, he investigates intuitive and necessary truths considered as motives to action, and, finally, terminates this portion of his subject by examining Mind regarded as the First Cause. This, one of the most interesting and suggestive arguments of Dr. Laycock's work cannot well be dealt with, so as to do justice to the author, in a brief analysis.

The first great division of Dr. Laycock's work ends with the doctrines of the Correlation of Causes. In the second division he applies the teleological doctrine to the phenomena of Life and Organization—to Biology. He discusses successively the fundamental conditions of existence considered teleologically; the laws of permanence with incessant change in organisms; the fundamental principles of morphology, or the laws of existence in relation to space; the law of evolutional development of archetypal forms; and the fundamental laws of vital action,—under this latter head examining (1) fundamental ideas as vital energies, and (2) the physiological correlations of the fundamental ideas.

Having completed the application of his method to biology, Dr. Laycock then proceeds to develope the principles of a scientific psychology.* And, first, he examines the question of the substratum of conscient mind, and the method of determining its laws in relation to consciousness. Starting from the fundamental proposition that mind as the first cause is inseparably associated with the primary forces of matter, and that it acts in and by those forces alone, he argues that the results of those forces in action are its exponents, and their phenomena are its signs or language, the expression of its thoughts. This which is true of the general laws of nature, is true also of the laws of life and organization; and, consequently, the phenomena of life and organization are to be regarded as true and manifest exponents of the laws of thought. We have seen that the vital forces, of which the so-called "nerve-force" is the type, and the physical forces are correlated; and since we can reduce the laws of the physical forces to numerical expressions, we ought to be able to reduce the laws of their derivative forces—those of life and organization—to numerical expressions—

"Now, this we are actually able to do in some degree as to those known as the laws of nutrition and development of organisms. We may, however, advance even a step further, and extend the method to the laws of life and organization in correlation with the laws of thought. For since all the successional states of our consciousness correspond to successional vital states, and since these occur according

* The subsequent references are to Vol. II. of Dr. Laycock's work.

to the law of the vital forces, it is clear that the signs which express the successional states of the one may be made to express or correlate the successional states of the other.

"It is very necessary, however, to bear in mind, in considering this question, that it is not changes in the mere matter of the brain in the concrete (as many are apt to think) which constitute the vital processes that are correlative with mental states; on the contrary, such changes taking place put a stop to the phenomena of thought, and often, indeed, of life. The changes we have to consider are dynamic, or teleorganic,* and are operated by those same vital forces by which the brain itself is built up, so that it shall, as a whole, be in express adaptation to the end that these successional states of consciousness should take place. In other words, the incessant vital changes which correlate thought do not differ in their nature from those which correlate growth, nutrition, and development.

"It is further to be carefully noted, that, as the vital forces correlate the physical forces, their laws of action will correlate the laws of action of the physical forces. Now if, in examining into these, the physicist has to distinguish between them and that substratum (whether atomic or not) in which they are hypothetically conceived to act, it is much more imperative on the physiologist and metaphysician to distinguish the forces with which he has to deal, from the substratum in which they also must hypothetically be conceived to act. These forces are put into action by what are really physical forces; for all the impressions made by mere matter or organisms are in fact vital changes induced by the physical force of matter: while all the impressions derived from living things, whether visual or auditorial, are equally made through the medium of those physical forces, and not otherwise. Every impression on the senses can be thus resolved finally into an impression of contact or touch; so that Touch is the fundamental sense, and all the organs of the senses are but very delicate instruments of touch. Such is the general law deduced from observation and experiment; such is the only law at which we can arrive *à priori*;† for since the vital forces are correlative with the physical, they can only be modified by those which correlate them." (pp. 8—10.)

The doctrine of psychical substrata was formally enunciated by Dr. Laycock fifteen years ago.‡ To those substrata, "the teleorganic changes of which hypothetically correspond to notional states of consciousness," he applied the term *ideagenic* substrata; to those "the teleorganic changes of which correspond hypothetically to motor phenomena," the term *kinetic* substrata.

After briefly indicating the bearing of the doctrine upon psychological inquiry, Dr. Laycock then enters upon the subject of

* A term first used by Mr. G. H. Lewes, to signify truly vital substances.

† Compare especially on this point, Sir William Hamilton, *Lectures on Metaphysics*, vol. ii. p. 152, *sqq.*; and Mr. H. Spencer, *Principles of Psychology*, part iii. chap. viii.; also Aristotle, *De Animâ*.

‡ See Dr. Laycock's paper *On the Reflex Function of the Brain*, in the volume of this Journal for 1845. For further development of the doctrine mentioned in the text, see also the vol. for 1855, p. 540.

the fundamental correlations of life and consciousness. One illustration from this portion of his work, in reference to feeling as a cause of adapted movements, must suffice.

"Now, there is a large number of purely vital movements of external relation as well as of internal, which manifestly show that a principle of adaptation to external things is in operation. In plants, that principle is identified by many with Life; in lower animals, with Instinct. But while, as to the former, it is not inferred that they indicate conscience or feeling in the organism, as to the latter it is confessed that they do,—and this on the ground that such movements in man and the higher vertebrates are associated with feeling. And since an act of energy accompanies these movements when pleasure or pain is felt, so that a desirable end is aimed at, it is concluded that the feeling is the cause of the adapted movements. But it is clear that this teleological theory ought to be equally applicable to the growth and movements of plants. Some of these, such as the "sensitive" plant, display motions of their parts strikingly similar to those of the lowest animal organisms. If, then, feeling be the assumed cause of the movements in the latter, why is it not the admitted cause of them in the former? To answer that they are plants, is simply to beg the question.

"But the theory of Plant-consciousness cannot, in fact, be either proved or disproved: yet whether it be granted or denied, the difficulties of the question are not thereby cleared up. It is quite certain that the simple embryo-cells of both plants and animals (zoospores and zoids) both move and act in adaptation to their external conditions, and that their organs are developed in relation to each other, so that they constitute a whole. But then, in various instances, as they are developed into what may be termed a higher phase, they lose their animal-like locomotive freedom, and become either plants or plant-like animals; so that if the embryo-cells have feeling, the full-grown organism is apparently unconscious. This is so wholly opposed to the order of events in the higher organisms, that if we admit these lower forms to be conscious, the conclusion is obvious, that their consciousness wholly differs from that of man." (pp. 23-24.)

Next in order of discussion come the correlations of the desires and feelings with the laws of vital action, immediately followed by an investigation of the general laws of evolution and manifestation of the mental faculties as active powers. This is followed by an inquiry into the fundamental correlations of the laws of growth and development and the laws of thought, in which the teleological method is applied with the happiest results to the laying of a firm foundation of a science of mental organology. In the first section of this subdivision of the work it is well reasoned that "without the mental causes operating as biotic ideas, there can be no development of a proper *substratum*; without the correlative *substratum* there can be no manifestation of mental powers, and no cognitions, whether primary or derivative" (p. 62); in the second section it is shown that "the laws of vital affinity must constitute the foundation of a true physiological psychology, just as the laws of chemical affinity constitute the foundation of a chemical science" (p. 66); in the third, the organismic evolution of new instincts and faculties in the species is inquired into; and in the fourth and last, the organismic degradation of instincts and faculties.

The faculties, feelings, and beliefs, as experience, are now systematically considered, and then the fundamental intuitions are treated as scientific ideas, or causal ideas of the sciences; and a general exposition of the unity of the laws of life and thought completes the portion of the work devoted to scientific psychology.

Dr. Laycock then endeavours to set forth the principles of a mental physiology developed according to the method which has already guided him to so many excellent results. This portion of his subject commences with an exposition of a doctrine that the functions of the encephalon are the seat of the unifying processes of life and thought. He writes :—

"Now, the first and most fundamental function of the encephalon is its unifying function. 1. It is the structure whereby the man is self-conscious—that is, mentally one. 2. But it is also the organ whereby he acts as one in relation to the external world; and, 3. It is equally the organ whereby all the processes of vegetative life are so influenced that the modes of vital activity reflect and coincide with the modes of mental activity. It is by this organ that the features, gait, tone of voice—*i. e.*, the *physiognomy*—proclaim the man's mental tendencies; it is equally by this that the vital and mental tendencies of the parent are impressed, as it were, upon the sperm-cell and germ-cell, and through these upon the primordial cell, out of which, in virtue of those tendencies, a new individual is evolved, bearing a specific and even individual resemblance to his parents, both as to form, function of organs, and mental character. Hence the encephalon is in relation, as a centre of union, with all corporeal processes whatever." . . . . (pp. 143-144.)

"If this view of the encephalic functions be correct, it is obvious that it is much more than the common centre of conscious activity, and that consequently those physiologists who have limited their inquiries to its relations to consciousness alone, or to the relations of its parts to a common centre or seat of consciousness—*i. e.*, a *sensorium commune*—have taken too limited a view of its function. For probably there is not any tissue of the body, nor the functions of any of its parts, which are not influenced directly or indirectly by this unifying energy of the encephalon. It follows, therefore, that in this structure we have the final evolution in organization of the fundamental teleiotic idea of unity, and that it is in truth a differentiation of the properties of all tissues whatever." (p. 145.)

Following this doctrine we have an historical review of researches into the law of cerebral differentiation with unity of function; and this, in turn, is succeeded by the principles and outline of a physiological classification of correlated mental and vital phenomena.—

> "It is only the teleiotic idea, which, being permanent, absolute, and universal, is sufficient for an arrangement which shall include both plant and animal organisms, and generalize the innumerable facts and observations upon which the ever-increasing naturalists' species and varieties are based." (p. 182.)

Dr Laycock rightly remarks that this doctrine, although not formularized, lies virtually at the bottom of Mr. Darwin's views upon the origin of species by means of natural selection.

The fundamental correlations of the instincts and appetites with vital motion occupy the next stage in the regular development of Dr. Laycock's scheme of mental physiology. After recapitulating the general laws of transference of vital force, he proceeds to show, by the application of the teleological method, the correlations of growth and visible motion, in development and reproduction, in the processes of aëration and alimentation, and in the conservative processes and instincts of external relation.

Dr. Laycock next questions the doctrine that the nervous system is the sole apparatus by which the functions of organs are co-ordinated, and the vital processes unified, and he examines the influence of the nutrient fluids and the blood as unifying and co-ordinating agents. He shows that the nutrient fluids of plants and animals which have not a nervous system, exercise a most important co-ordinating function. He points out how, in the higher animals, the circulatory system advances *pari passu* in its differentiation with the evolution of the blood-corpuscle, and of a more complex nervous system. He traces the teleiotic idea which is developed in the entire blood-distributing apparatus, and the functions of the capillary walls, and argues that it is the fundamental idea of the blood-corpuscle itself, so that the contractile function of the cell-wall, and the motor powers of the capillaries may be looked upon as derivatives of "that law of vital affinity between the living tissue and its contents, which is the general law of vital motion." Further, when nerves accompany vessels, he holds that their function is to be regarded as "only a higher evolution of the same general law, and is necessary to secure more effectually the unity of function and relation to all the multiform elements of the organism, that have to co-operate in action to the ends of its existence."—(p. 25.) It would follow, therefore, from this doctrine, that although the functions of an organism "may be duly performed without any nervous system, yet, when the nutrient fluid is more highly differentiated into what we call blood, then all the processes which primarily depend upon it, such as secretion, excretion, growth (or multiplication of cells), and the vegetative processes generally, are harmonized or co-ordinated by the nerves, through their influence on the motor powers of the blood-distributing capillaries."—(p. 226.) Pursuing this notion, Dr. Laycock, in a subsequent paragraph, writes :—

"It cannot be doubted, therefore, that while there is an intimate relation on the one hand between the blood-vessels and the blood, and on the other hand between the blood-vessels and the nerves, there is an important correlation of function between the blood and the nerve. The blood is, in truth, a body of floating cells coming into continual contact with the histological elements of the nerves and nerve-centres, in common with those of all other tissues, as it circulates through them. Now, considered from this point of view, the blood is, in fact, situate externally to the nerve-cells, just as the nutrient flood is in contact with the surface of the endoderm in the polype; and as they are the tissues in which the highest specialization of that law of adaptation which is manifested generally in the polype, it is obvious that their function in relation to the blood will be characterized by the highest adaptation to the ends of the organism. Hence the nervous system may be looked upon as an immense co-ordinating apparatus, the function of which, in relation to the blood, is to maintain the integrity and fitness of the nutrient fluid, to distribute it fitly to all the manifold differentiations of organs, and to subserve to the proper deposit from it of all those elements which are required to be deposited in differentiated structures, whether in the course of nutrition, growth, repair, or excretion."

Dr. Laycock now commences an investigation of the correlation of the sympathies and antipathies with the primordial instincts, and, having completed this, passes on to an examination of the correlations of the communistic instincts with the fundamental laws of differentiation and integration in morphology and development. The treatment of this latter subject may be judged of by the terminal paragraph devoted to it.

"The entire class of facts detailed in this chapter points conclusively to the general principle, that the consciousness in the entire series of animals, from the Invertebrata downwards, probably differs in kind from that of man and the vertebrates generally. Nevertheless we can conclude from them that the great laws and needs of human society do not arise capriciously, but that they have their correlatives in the deepest and most primary manifestations of the laws of life and organization. The highest evolution of what I have termed the Primordial Instincts is seen in the communistic instincts of the two classes of animals which are at the head of their respective archetypal branches; viz., the social insects, the most highly developed of the Invertebrata; and the social man, the most highly developed of the Vertebrata; in both, the family instincts constitute the solid foundation of society. Hence it is that in proportion as they are active in a nation, in the same proportion its social organization is vigorous and complete."

The physiology of the corporeal feelings and desires, of the appetites, and of the corporeal loathings and aversions, is next discussed, and an examination of the relations of the vital powers to mental character and manifestations completes the section of the work devoted to mental physiology.

Throughout the whole of the sections on scientific psychology and mental physiology, practical applications and suggestions (of which we have barely given an occasional hint in the course of our brief outline) abound, and the interest of the work never flags.

The last portion of Dr. Laycock's treatise is devoted to the principles of Mental Organology. The general functions of the nervous system are, first, most felicitously treated, and the fundamental ideas which are evolved in the functions are traced. The nervous system is regarded not only as an apparatus "whereby the causal changes subserving to adopted motion and to consciousness shall take place," but also as "an apparatus whereby the changes which occur everywhere in the body are unified."

"The forces resulting from the nervous system are looked upon as in the nature of a dynamical stimulus and vital changes, in virtue of which the forces requisite thereto are evolved; just as a transference of force from matter, in the shape of an external affinitive impression is a dynamical stimulus. Hence the so-called *vis nervosa*, transmitted from the ganglionic centres, is an affinitive stimulus to fitting changes in the tissues—*i. e.*, the adapted production and transference of force, but not necessarily the force itself by which these changes are effected. It is therefore analogous to the forces of heat, light, chemical affinity, and the like, which when received directly from appropriate things, as affinitive impressions, by the living tissues themselves, excite various changes therein. The difference, therefore, is, that where there is a nervous system, these affinitive impressions, coming through appropriate organs of reception, act first upon its co-ordinating mechanism, and through it upon the tissues. Now the fundamental co-ordinating apparatus is probably the nerve-cell." (p. 331.)

The general homologies and anatomy of the nervous system are next considered, and then Dr. Laycock enters upon an examination of its dynamic and structural elements. The evolution of the nervous system in development and function is also considered, after which the functions of the sympathetic and intervertebral, and the organology of the cerebro-spinal systems are investigated.

It would be impossible to convey in a brief form a just notion of the final portion of Dr. Laycock's work. His examination of the functions, evolutions, organology of the nervous system, whether it be regarded simply as a summary of our knowledge on these subjects, or on account of its suggestiveness, is in the highest degree interesting. He throws much light upon the reciprocal relations of the different divisions of the nervous system and upon the functions of several of its most important centres, particularly of the spinal cord and medulla oblongata. He places, indeed, the whole of our knowledge of the nervous system in a clearer light and upon a firmer foundation, and his conclusions and suggestions are admirably adapted to facilitate, and to give an additional impetus to, further research.

We have now completed our description of this important work, but any description would fail to impart an adequate notion of the excellent philosophy which characterizes it, and of the rare ability with which the method of research the author has adopted is carried out and sustained over a vast field of observation. How happily the work supplies the need we referred to at the commencement of this article must, however, we think, be apparent to our readers from what we have already said; and no additional observations can be required to show that the book also possesses, in an eminent degree, that soundmindedness and capability of fostering psychological and biological research, which we have set down as the criterions by which we should judge of it as a class-book for students. We must add, in conclusion, that Dr. Laycock's treatise is one which is alike of importance to the philosophical physician, the physiologist, and the psychologist, and its publication, we trust, will mark an epoch in the psychological literature of this country.

---

